# Opportunities, Challenges, and Scientific Progress in Hemp Crops

**DOI:** 10.3390/molecules29102397

**Published:** 2024-05-20

**Authors:** Kacper Piotr Kaminski, Julia Hoeng, Fernando Goffman, Walter K. Schlage, Diogo Latino

**Affiliations:** 1 Independent Researcher, 2000 Neuchâtel, Switzerland; 2 Vectura Fertin Pharma, 4058 Basel, Switzerland; 3 Seedcraft S.L., 30510 Yecla, Spain; 4 Independent Researcher, 51429 Bergisch Gladbach, Germany; 5 Independent Researcher, 6300 Zug, Switzerland

**Keywords:** hemp, cannabis, cannabinoids, cannabidiol, CBD, terpenes

## Abstract

The resurgence of cannabis (*Cannabis sativa* L.) has been propelled by changes in the legal framework governing its cultivation and use, increased demand for hemp-derived products, and studies recognizing the industrial and health benefits of hemp. This has led to the creation of novel high-cannabidiol, low-Δ^9^-tetrahydrocannabinol varieties, enabling hemp crop expansion worldwide. This review elucidates the recent implications for hemp cultivation in Europe, with a focus on the legislative impacts on the cultivation practices, prospective breeding efforts, and dynamic scientific landscape surrounding this crop. We also review the current cultivars’ cannabinoid composition of the European hemp market and its major differences with that of the United States.

## 1. Introduction

### 1.1. History and Perspective of Hemp Cultivation in Europe

Hemp is undoubtedly one of the most important crops cultivated in human history and was one of the first plants cultivated in Europe [1]. In Europe, hemp has a rich history of traditional uses that date back centuries. Commonly cultivated for its strong fibers, which were used to produce textiles, ropes, and sails, hemp was a valuable commodity in maritime industries and everyday life. Additionally, hemp seeds were used as a food source and in traditional medicine for their nutritional and therapeutic properties [2].

The history of *Cannabis* genus plant laws in Europe has been intricate and diverse, as each country adopted distinct approaches and regulations concerning the cultivation, possession, sale, and use of cannabis. In the early 20th century, when international drug control efforts began to take shape, numerous European countries enacted laws to regulate the use of all *C. sativa* varieties [3]. The International Opium Convention of 1925 was the first international treaty that addressed cannabis control, defining ‘Indian hemp’ as the dried flowering or fruiting tops of the pistillate plant *C. sativa* from which the resin has not been extracted, regardless of its commercial designation. Many European nations subsequently implemented national laws to comply with this treaty. At that time, low-Δ^9^-tetrahydrocannabinol (THC) plants (hemp) were regulated to the same extent as high-THC varieties (marijuana, more recently known as ‘medical cannabis’) of cannabis [4].

In 1961, the United Nations’ Single Convention on Narcotic Drugs was introduced, aiming to control the production and distribution of cannabis and other narcotic drugs [3]. This had a significant impact on the development of stricter drug policies in European countries. In the United States, there were already laws in place restricting the cannabis industry, predating the Single Convention on Narcotic Drugs [5]. As global efforts to restrict drugs gained momentum, many European nations adopted stringent drug laws, criminalizing the possession, cultivation, and distribution of cannabis plants [6].

However, in the latter part of the 20th century and into the 21st century, some European countries began to decriminalize possession of small amounts of cannabis for personal use, treating it as an administrative, rather than a criminal, offense [7]. In response to growing evidence of the therapeutic benefits of cannabis, several European countries started legalizing medical cannabis in the 21st century [8,9].

### 1.2. Past and Future of European Hemp Science and Applications

Cannabis (*Cannabis sativa* L.) has a long history of use in various parts of the world, including Europe. Its fibers and seeds have been used traditionally for many purposes, including clothing, building materials, paper, nutrients, and human and animal health [1,2]. Because of psychotropic use in the 1920–1930s of the high-Δ^9^-tetrahydrocannabinol (THC) content-dominant varieties, cannabis was largely banned in most countries in subsequent decades, including in most of Europe [1]. However, legislators now recognize the difference between high THC-containing plants (i.e., marijuana) and hemp, which has a low THC content, and is, therefore, non-euphorigenic and an excellent source of beneficial cannabinoids, such as cannabidiol (CBD) [10]. Taxonomically, it is thought that both marijuana and hemp belong to a single species, *C. sativa* L., which encompasses all cannabis/hemp varieties [11]. However, based on observations of phenotypic differences, some scientists believe that the *Cannabis* genus comprises three species, namely *C. sativa* (hemp), *C. indica* Lam (marijuana), and *C. ruderalis* [12,13]. The original classification of cannabis varieties according to their phenotype distinguished three types: Type I—THC-dominant, Type II—THC/CBD-balanced, and Type III—CBD-dominant [14]. Later on, another chemotype was identified and described and has since been called Type IV— CBG-dominant [15]. Only in very recent research was the final type added to the chemotype classification, Type V, which accumulated few if any cannabinoids [16]. In this review, we will focus on low-THC *C. sativa* hemp varieties, which have enjoyed a noticeable surge in cultivation in recent years because of increasing interest in consumer and medical applications across Europe [2].

### 1.3. CBD Promotes Revival of Hemp Cultivation

In recent years, hemp has experienced a remarkable revival, primarily because of its emergence as a valuable source of cannabinoids other than the psychotropic THC, particularly CBD. This resurgence marked a significant shift from the historical stigmatization of cannabis, as it became recognized that the low-THC plant (hemp) could be utilized for numerous industrial applications [17] and for the therapeutic potential of its cannabinoid-rich extracts [18,19]. Specifically, the medicinal potential of hemp is primarily associated with its cannabinoid content, particularly CBD [20]. CBD has been widely studied for its potential in alleviating various health conditions, including pain [21,22,23,24], anxiety [25,26], epilepsy [27,28], and inflammation [29,30].

There has been a notable surge in the development of hemp inhalation studies [31,32,33,34], driven by the increasing interest in hemp-derived compounds and the growing acceptance of alternative methods of consumption. These products primarily focus on delivering cannabinoids such as CBD through various inhalation techniques, including vaporization and smoking [35,36,37,38]. Innovations in vaporization technology have led to the creation of portable and user-friendly hemp vaporizers, allowing consumers to inhale CBD-infused vapors without the harmful byproducts associated with traditional smoking. Additionally, hemp pre-rolls, containing high-CBD hemp flowers, have gained popularity for convenient and discreet hemp consumption through smoking. These products offer consumers a quick onset of effects and are favored for their potential therapeutic benefits, making them a prominent segment in the expanding hemp market.

Some hemp varieties are naturally abundant in CBD, making them an appealing option for producing CBD products without the unwanted psychoactive THC effects associated with marijuana. This has led to a surge in hemp cultivation worldwide, with farmers and entrepreneurs seeking to capitalize on the growing demand for CBD-driven health and wellness properties [39,40,41,42]. However, the appeal of hemp extends beyond CBD. The plant can produce over 140 cannabinoids in total [43,44], among them, a variety of non-psychoactive, biologically active cannabinoids, such as CBG [45,46,47,48], cannabichromene [49,50,51], cannabinol (CBN) [52,53,54,55], cannabidivarin (CBDV) [56,57,58,59], and tetrahydrocannabivarin (THCV) [60,61,62,63], with each of them exhibiting potential health benefits. Scientists are increasingly exploring the therapeutic properties of various cannabinoids to better understand their potential health applications.

### 1.4. Industrial Versatility of Hemp

Hemp fibers have been used for millennia in the production of textiles and fabrics. Hemp fabrics are known for their durability, breathability, and resistance to UV rays. Hemp textiles are not only environmentally friendly but also offer a sustainable alternative to traditional cotton [64,65] and synthetic fibers [66,67].

Hemp-based construction materials, such as hempcrete, have gained popularity as eco-friendly alternatives in the building industry [68]. In several studies, hempcrete performed well as a building material, replacing traditional construction materials while adhering to the thermal, insulating, and acoustic characteristics required in construction [69,70,71,72,73].

Hemp seeds are rich in essential nutrients and have gained recognition as a superfood. They are a complete source of protein, containing all nine essential amino acids, making them an ideal plant-based protein option for vegetarians and vegans [74,75,76]. Hemp seeds are also abundant in healthy fats, particularly omega-3 and omega-6 fatty acids [77], promoting heart health and overall well-being [78].

Hemp seed oil is a prized ingredient in the cosmetic and skincare industry because of its nourishing and moisturizing properties [79]. It is a natural emollient, helping to soothe and hydrate the skin without clogging pores. Hemp seed oil is also rich in antioxidants [80], aiding in the fight against free radicals and supporting skin health [81]. It can also be used as an anti-inflammatory agent, particularly in irritable bowel syndrome and other gastrointestinal conditions [82]. Hemp seed oil can also be used as a biofuel [83,84]. Hemp biodiesel has shown promise as a renewable and environmentally friendly alternative to fossil fuels [83,84]. CBG is a cannabinoid found in cannabis with potential health benefits. Research suggests CBG might have neuroprotective properties, anti-inflammatory effects useful for disorders such as inflammatory bowel disease, and potential pain relief capabilities. Additionally, CBG could aid in stimulating appetite and possibly serving as an antimicrobial agent [47]. In another study beneficial effects on anxiety, chronic pain, depression, and insomnia were reported with few if any side effects [85].

To summarize, hemp exhibits great promise as a sustainable alternative to traditional materials and crops. Its versatility, carbon sequestration capabilities [86,87], and moderate growth cycle make it an attractive option for numerous industries seeking eco-friendly solutions. In Malawi, where hemp is replacing tobacco cultivation, the use of hemp has led to reduced water consumption and pesticide use, contributing to a more sustainable agricultural and industrial landscape [88,89]. As awareness of the environmental benefits of hemp continues to grow, more regions and industries may consider incorporating this versatile plant into their practices, fostering a greener and more sustainable future.

## 2. Major Hemp Compounds and Breeding Efforts

### 2.1. Exploring Major Cannabinoids

The most important compounds of *C. sativa* are cannabinoids, with THC being the most well-known one because of its strong psychoactive effect, while the second most-known one and target of multiple breeding efforts is CBD [90]. However, the cannabis plant produces more than 140 different cannabinoid compounds [11,12,90]. We will not describe the detailed biosynthesis or the chemical transformations of cannabinoids, as such processes have been extensively described elsewhere [91,92]. Substantial clinical evidence exists for the efficacy of CBD in the settings of anxiety, psychosis, schizophrenia, post-traumatic stress disorder, and substance abuse [20]. This includes uncontrolled and randomized controlled trial (RCT) studies for anxiety, psychosis, schizophrenia, post-traumatic stress disorder, substance abuse, and sleep quality [20].

Regarding biological activity, it is important to consider the stereoisomers of Δ^9^-THC, which has two stereogenic centers (C-6a and C-10a) and can exist as pairs of enantiomers and diastereomers (two enantiomers of Δ^9^-trans-THC and two enantiomers of Δ^9^-cis-THC) [93]. It has been shown that low-THC hemp varieties are rich in Δ^9^-cis-THC in concentrations comparable with that of Δ^9^-trans-THC, which is predominantly responsible for psychoactive effects [94]. The enantiomers of Δ^9^-cis-THC had less CB1/CB2 binding (Ki) and functional activity (EC50 [35S]GTPγS binding) for the inhibition of the endocannabinoid-degrading enzymes (IC_50_ values) than (−)-Δ^9^-trans-THC in a comparative in vitro biological evaluation [94].

### 2.2. Understanding Terpenes

Multiple terpenes are found in cannabis plants, where they are abundant and complex in nature, contributing to the overall aroma and scent of different varieties and are a major breeding focus [95]. Terpenes are responsible for the distinct aromas and flavors associated with various plants, fruits, and herbs, but more importantly, they play essential roles in the plant kingdom, serving as a defense mechanism against herbivores and pathogens [96,97], as well as attracting pollinators [98].

The most prevalent terpenes in cannabis are myrcene, which is recognized for its musky, earthy, and fruity fragrance and linked to relaxant, sedative, anti-inflammatory, and analgesic effects [99]; α- and β-limonene, which emits a citrusy, lemon/orange-like aroma and is associated with mood elevation and stress alleviation [100] and neuroprotective properties [101]; α-pinene, which elicits a piney scent akin to coniferous trees, potentially possesses anti-inflammatory properties [102], and aids brain health [103]; β-caryophyllene, which has a peppery aroma such as that found in black pepper and cloves, with neuroprotective [104] and antioxidant properties [105]; linalool, which is known for its floral, lavender-like aroma that is connected with relaxation and stress reduction [106]; humulene, which exudes an earthy, woody scent, and is being researched for its potential anti-inflammatory capabilities [107]; terpinolene, which features a complex bouquet of floral, herbal, and citrus notes, though it is less common and its effects remain under scrutiny [108]; ocimene, with its sweet, herbal, occasionally fruity fragrance, which is believed to have antiviral and antifungal properties; nerolidol, which is characterized by a woody, citrusy aroma and undergoing exploration for potential anti-parasitic and antimicrobial benefits [109,110]; and α-bisabolol, which emits a sweet floral aroma and is under investigation for potential pharmacological effects [111].

Some studies have indicated that terpenes neither act on cannabinoid receptors directly [112] nor activate transient receptor potential vanilloid 1 and ankyrin 1 channels nor modulate their activation by THC [113]. However, other studies have shown that terpenes can activate the CB1 receptor in vivo [114]. Cannabis terpenes α-humulene, geraniol, linalool, and β-pinene have also been shown to activate the CB1 receptor in vivo and can in fact be multifunctional cannabimimetic ligands [115]. Although the action of terpenes on cannabinoid receptors is unclear, they have gained significant attention in the cannabis industry and the broader field of aromatherapy and natural medicine because of their potential health benefits [116]. Different terpenes may exhibit various effects, such as promoting relaxation, reducing stress, improving focus, or providing anti-inflammatory properties.

Cannabis contains other aromatic compounds, such as aldehydes, ketones, alcohols, esters, nitrogen-containing compounds, and phenols, which contribute to its characteristic aroma and flavor [116,117]. These aromatic compounds work in synergy with terpenes to create the diverse and complex scents found in different cannabis varieties [116]. The presence and concentration of these compounds can vary among varieties, giving rise to the wide range of aromas associated with various cannabis varieties [116].

Phenolic compounds encompass a range of aromatic compounds with pivotal roles in both plant defense mechanisms and potential human health benefits [116]. Some prominent phenolic compounds found in cannabis include flavonoids such as quercetin, kaempferol, luteolin, and apigenin [118]. Quercetin, a flavonol, has been detected in noteworthy concentrations and is recognized for its antioxidant and anti-inflammatory attributes [119]. Similarly, kaempferol, another flavonol, contributes to the antioxidant capacity of cannabis [120] and holds promise for its potential cardioprotective effects [121]. Apigenin, a flavone, is known for its anxiolytic properties and potential as an anti-inflammatory agent [122]. Furthermore, cannabis also contains cannflavins A and B, unique flavonoids with emerging research highlighting their potential anti-inflammatory properties [118]. These phenolic compounds, along with others, underpin the intricate biochemical profile of cannabis, potentially influencing its aroma, flavor, and therapeutic potential. Although the precise roles and interactions of these phenolic compounds are still being elucidated, their presence underscores the multifaceted nature of cannabis and its potential applications in both plant biology and human well-being.

Among the more interesting recent discoveries was the identification of a new family of volatile sulfur compounds containing the prenyl (3-methylbut-2-en-1-yl) functional group that is responsible for skunk scent [123]. Their remarkable similarity to garlic volatile sulfur compounds also marks them as a target for discovery of their additional health benefits [123].

### 2.3. Advancing Classical Cannabis Breeding

Since the first sequencing effort to produce the draft genome of *C. sativa* [124], there have been many subsequent studies improving genome assembly and expanding on the number of varieties [125,126], with the most recent and well-regarded one being published in 2021 [127].

Geographical expansion and domestication have had little impact on the *Cannabis* genome size. Although there are significant differences between male and female genome sizes, they cannot be distinguished via combined flow cytometry [128], which is useful for the hemp industry as it is female plants that produce flowers with desired cannabinoids. and identification is now easily achieved by testing for Y chromosomes using PCR [129].

There have been recent efforts in Europe to develop improved methods, such as rapid generation cycling (speed breeding), to produce hemp varieties adapted to local climate conditions and specific applications [130]. Hemp is inherently a short-day plant requiring 12–14 of daylight hours for optimal growth. While longer days promote yield and reduce flowering, these are suitable for the production of hemp as a fiber source. It remains unclear if the performance of hemp varieties grown in higher-latitude regions will hold true in lower-latitude and tropical regions [131]. On the other hand, longer dark periods can induce early flowering and reduce biomass. Other efforts have focused on developing improved protocols for rapid regeneration, such as modified nodal cutting and shoot-tip protocols [132] or seed priming and pericarp removal [133]. Further studies have been undertaken to measure cannabinoid concentrations and their correlation with climate conditions [134].

It is important to note that cannabis offers remarkable plasticity and is perfectly suitable for breeding efforts that follow Mendelian selection. In addition to classical methods, the combination of breeding and genetic engineering, including CRISPR technology, hold immense potential for optimizing hemp plants to maximize specific cannabinoid and terpene contents. Genetic engineering, particularly the precise and targeted modifications facilitated by CRISPR, offers the ability to directly manipulate the *Cannabis* genome [135,136]. This advancement allows scientists to enhance the expression of genes responsible for cannabinoid and terpene production or introduce novel pathways to produce rare or valuable compounds [137,138,139]. By harnessing these techniques, hemp cultivators can develop varieties tailored to meet specific market demands for medicinal, recreational, or industrial purposes, unlocking the full potential of this versatile plant. However, ethical considerations, regulatory compliance, and responsible research practices are crucial in exploring the potential of breeding and genetic engineering to ensure the sustainable and safe development of optimized hemp varieties.

In recent years, there has been growing interest in the biosynthesis of minor cannabinoids, which are cannabis compounds present in lower abundance compared with major cannabinoids, such as THC and CBD [140,141,142,143]. Research in this field has sought to elucidate the enzymatic pathways involved in the synthesis of these minor cannabinoids and explore the genetic factors influencing their production [143]. Scientists have identified various biosynthetic pathways responsible for minor cannabinoids, such as CBG [144], CBN, cannabichromene, cannabidivarin, and tetrahydrocannabivarin [145]. This knowledge has led to advancements in biotechnological approaches, including genetic engineering and synthetic biology, to enhance the yield and accessibility of these compounds [137]. The exploration of minor cannabinoids holds promise for their potential therapeutic benefits, as early studies indicate that some of these compounds may possess unique medicinal properties. As the field of cannabis research continues to expand, further understanding of the biosynthesis of minor cannabinoids may unlock exciting opportunities for the development of novel pharmaceuticals and therapeutics.

The accumulation of cannabinoids in cannabis plants is influenced not only by genetic factors but also by environmental conditions [145,146,147,148]. The genetic makeup of a cannabis variety determines its potential to produce specific cannabinoids [145,149], but environmental factors play a vital role in whether these traits are expressed to their fullest. Elements such as temperature, light intensity, humidity, soil composition, and nutrient availability impact cannabinoid accumulation [146]. For example, some varieties may produce higher THC levels under specific light conditions [147], whereas others may favor CBD production in different environments. Additionally, stressors such as drought [150] or pest attacks can trigger the production of certain cannabinoids as a defense mechanism [151]. This intricate interplay between genetics and the environment highlights the importance of understanding and optimizing cultivation practices to achieve desired cannabinoid profiles in cannabis plants [152]. By controlling environmental variables, cultivators can maximize the expression of desired cannabinoids, tailoring cannabis varieties to suit various consumer preferences and specific end-use applications.

## 3. The Growing Hemp Industry in Europe

In the European Union (EU), hemp cultivation is governed by regulations that define hemp as *C. sativa* having a THC content below 0.3%. Hemp farmers must exclusively use certified seed from varieties listed in the EU Common Catalogue of Varieties of Agricultural Plant Species. The Plant Varietal Portal of the European Union provides a searchable catalog of such EU-approved hemp varieties through its website [153]. Cultivators must also obtain licenses or authorizations, and regular inspections are conducted to ensure compliance with the THC limit. Additionally, regulations cover the processing and trade of hemp-derived products, such as CBD extracts, which may require further licensing or authorization.

**Figure 1:**
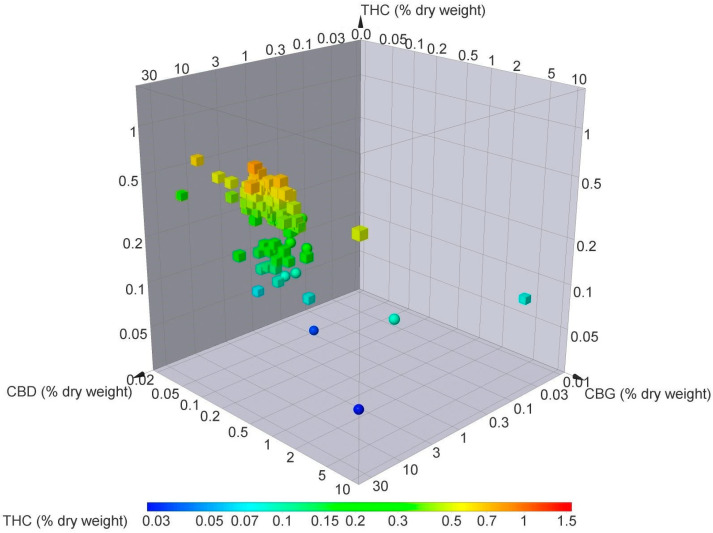
Three-dimensional chart of European Union and Swiss varieties of *C. sativa* L. Concentrations of THC, CBD, and CBG contents as percentage (%) of dry weight.

In Switzerland, farmers are required to obtain permits for legal hemp cultivation, and the THC limit is set at 1.0%. Switzerland also regulates the processing and trade of hemp-derived products to ensure compliance with the established guidelines [154]. Contrary to the EU, farmers in Switzerland are permitted to cultivate and trade any hemp variety regardless of origin if it possesses a THC content below 1.0% (Figure 1).

Several countries and regions in Europe have become prominent players in hemp cultivation [155,156]. France has a longstanding history of hemp farming and a well-established industry serving both domestic and international markets [157]. Germany has seen notable growth due to increasing demand for hemp products and supportive government policies. Italy’s favorable climate has contributed to its thriving hemp industry. Furthermore, Spain, particularly in regions such as Andalusia and Catalonia, has experienced significant expansion of cultivation [158,159]. The Netherlands, with its progressive approach to cannabis-related industries, has been actively involved in hemp farming for various applications. Moreover, Eastern European countries, including Poland [160], Romania, and Lithuania, are emerging as key contributors to the European hemp sector, witnessing growing cultivation areas and processing facilities [155]. However, it is necessary to consider the critical importance of maceration and the availability of water resources in hemp processing, especially given the diverse environmental conditions in many regions where hemp cultivation takes place.

The higher THC threshold of 1.0% enables the production of a wide range of high-CBD hemp varieties, contributing to a country’s flourishing market for CBD products [161,162]. While Swiss farmers benefit from diverse microclimates [163] that support the cultivation of various hemp varieties [164], ultimately, low humidity, long growing season, and high solar radiation that will best aid CBD-dominant varieties.

Hemp cultivators in Europe encounter a range of challenges and opportunities. One of the major hurdles lies in the diverse regulations across European countries concerning hemp cultivation, THC limits, and hemp-derived product processing, leading to uncertainty and hindering cross-border trade [165]. The stigma associated with cannabis, despite hemp’s legal distinction from marijuana, can impact investment and market acceptance. Competition from other agricultural sectors and imported hemp products further adds pressure to local cultivators.

Nonetheless, there are promising prospects for hemp cultivators in Europe. The increasing demand for hemp-derived products, especially CBD, presents a vast market potential for farmers and processors. As consumers gravitate toward sustainable and eco-friendly alternatives, the appeal of hemp-based goods increases significantly. The industry’s growth is supported by increasing research and innovation, leading to improved cultivation practices and the development of high-performing hemp varieties. Collaborative efforts between European countries can harmonize regulations, facilitate cross-border trade, and expand market reach for hemp cultivators.

## 4. THC- vs. CBD-Dominant *Cannabis sativa* L. Varieties

**Figure 2:**
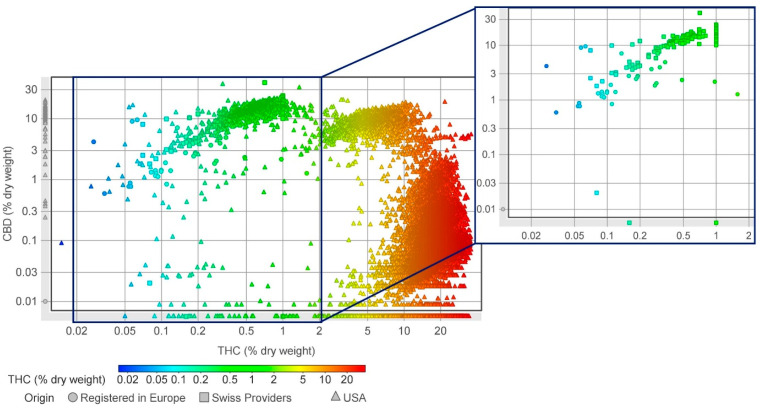
Concentrations of THC and CBD in cannabis varieties in the United States, Europe, and Switzerland. Data sourced from producers’ Certificates of Analysis as well as scientific publications. The full data are available as Supporting Table S1. Varieties in Europe are represented by circles, those in the Switzerland by squares, and those in the USA by triangles. Additionally, the THC concentration of each variety is shown in blue-red scale. An enlarged image of the European and Swiss varieties is provided on the right side of the figure.

**Figure 3:**
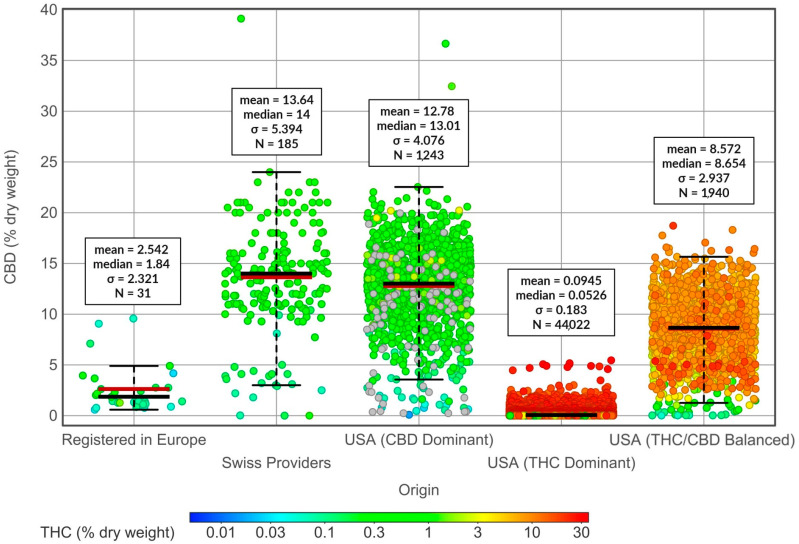
CBD concentrations (% of dry weight) in cannabis varieties in the United States, Europe, and Switzerland. Respective total cannabinoid concentrations are represented by red-blue color code (% dry weight). Mean, median, standard deviation, and number of varieties are indicated in boxes next to respective chart cloud bars.

The cannabis flowers from the USA and Europe contrast in their botanical features and chemical composition (Figure 2 and Figure 3). USA varieties have a significantly higher THC concentration than those from Switzerland or the rest of Europe, which have a much lower CBD content on average, particularly when compared with Swiss varieties (Figure 2 and Figure 3).

It is worth noting that the legislative limit of 0.3% THC in Europe highly influences the amount of CBD content (mean = 2.542%); the limit of 1.0% THC in Switzerland has no such effect (CBD mean =13.64%) (Figure 3). This relationship between THC content limits and CBD content is particularly evident when comparing CBD content in Swiss varieties with that of CBD-dominant varieties in the USA, where no THC limit is enforced (CBD mean = 12.78%) (Figure 3). Cannabis flowers from the USA, often characterized by their lush and resinous appearance, tend to have higher THC levels, which contributes to their potency and psychoactive effects [166,167]. Cultivated primarily as a source of fiber and seed oil, European cannabis flowers are CBD-dominant, offering potential therapeutic benefits without inducing psychoactive effects. The varying climate and regional cultivation practices in each continent contribute to the unique characteristics of their respective cannabis flowers, offering diverse varieties and effects.

**Figure 4:**
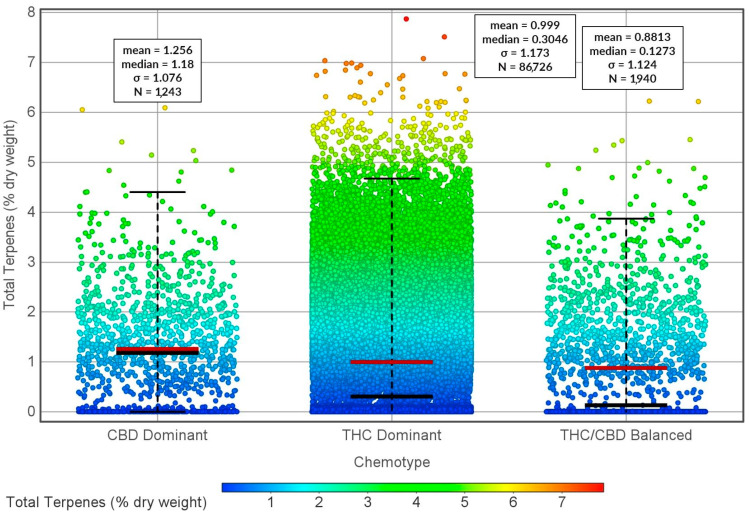
Concentrations (% of dry weight) of total terpenes in THC-dominant, CBD-dominant, and THC/CBD-balanced cannabis varieties in the United States.

**Figure 5:**
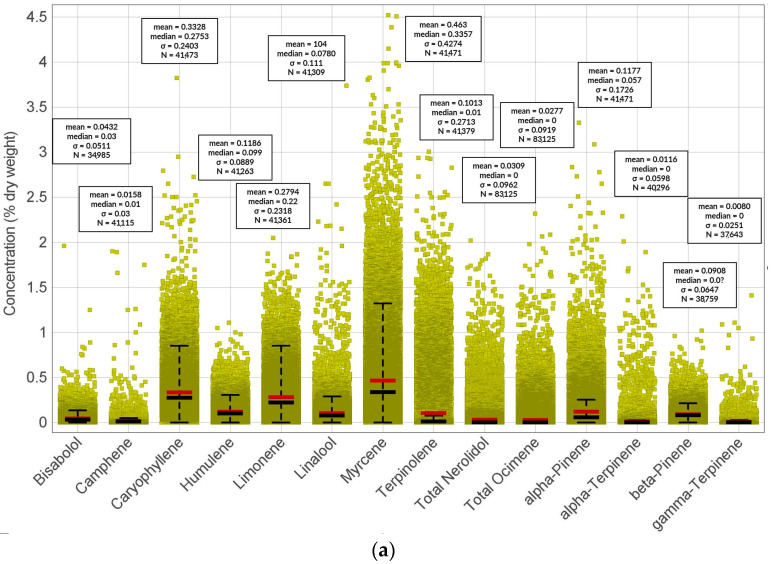

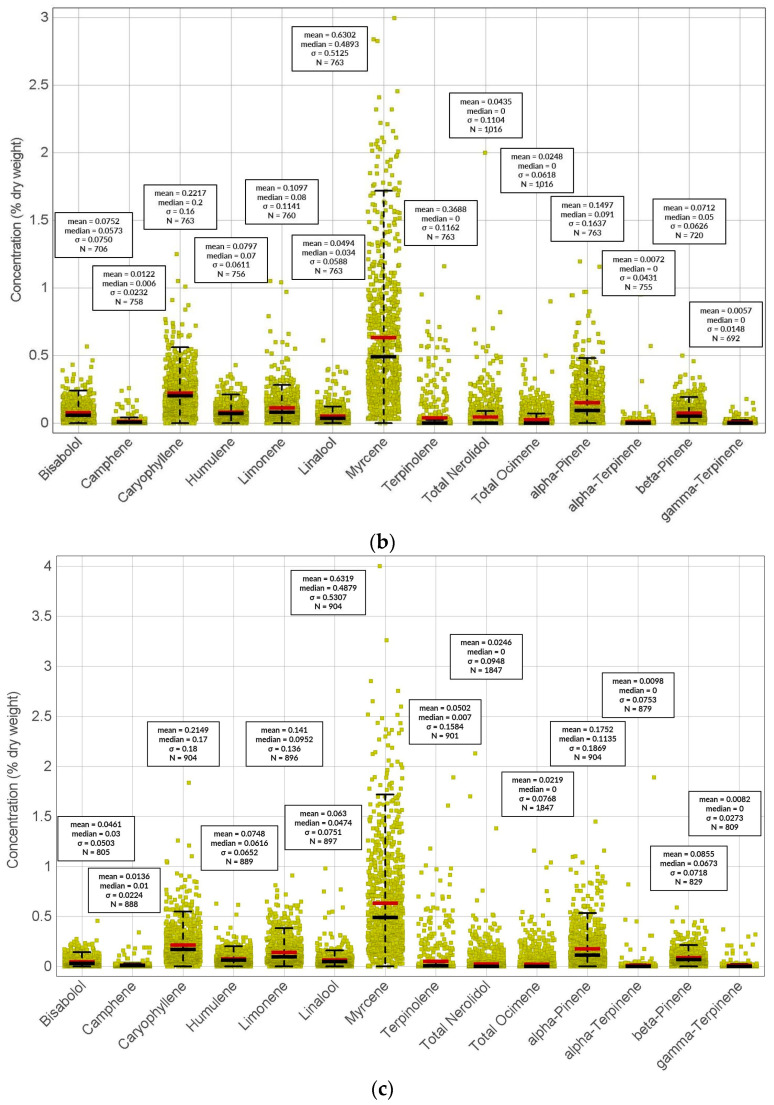
Concentrations (% of dry weight) of particular terpenes in (**a**) THC-dominant, (**b**) CBD-dominant, and (**c**) THC/CBD-balanced cannabis varieties.

In THC-dominant cannabis varieties, the types and concentrations of terpenes present can differ from those in CBD-dominant varieties (Figure 4 and Figure 5). The number of total terpenes does not significantly differ among CBD-dominant, THC-dominant, or CBD/THC-balanced varieties (Figure 4). This could suggest independent terpene accumulation and biosynthesis from that of determining cannabinoid dominance. When comparing particular terpenes among CBD-dominant, THC-dominant, and CBD/THC-balanced varieties, no clear trend emerges that would favor any terpene pattern in relation to specific cannabinoid accumulation (Figure 5). These terpene variations contribute to the distinct aromatic profiles and potential effects of the two types of cannabis.

However, the CBD-dominant strains of cannabis exhibit a greater degree of consistency in terpene profiles in comparison with their THC-dominant counterparts (Figure 5 standard deviations). This divergence in terpene variability can be attributed to the fact that a narrower range of CBD-focused varieties have been cultivated in contrast to the broader array of THC-dominant cultivars. This phenomenon is primarily a result of prevailing consumer inclinations within the United States market [167]. Recently, endeavors in the realm of breeding are being channeled toward the augmentation of aromatic and flavor profiles within CBD-dominant cultivars, specifically hemp.

In the hemp industry, significant discrepancies exist between reported and measured cannabinoid concentrations in various hemp products, posing challenges to consumers and businesses alike [168]. This disparity arises from a combination of factors, including the absence of standardized testing methods and regulations for cannabinoid analysis [169]. Different laboratories employ varying testing protocols and equipment, leading to inconsistent results for the same product. The inherent variability in cannabinoid composition within the same hemp product is another key factor influencing reported concentrations [170,171]. Factors such as plant genetics, growing conditions, and extraction methods contribute to this variability.

Additionally, the decarboxylation process, where raw cannabinoids are converted from their acid form in the plant into their pharmacologically active forms, can impact the reported cannabinoid content. Incomplete or non-uniform conversion during decarboxylation may lead to discrepancies between the reported levels of cannabinoids and their actual concentrations in the final product. Furthermore, inaccurate labeling practices and differences in extraction efficiency and sample preparation techniques can also contribute to the variation in reported cannabinoid concentrations [172,173,174,175]. It is also important to consider the degradation of cannabinoids, once formulated, in products and the limited shelf life resulting from this process [176,177,178].

To address these challenges and enhance consumer confidence, the European hemp industry needs standardized testing protocols and clear regulations for cannabinoid analysis. Implementing third-party testing and certification programs can provide reliable information on cannabinoid content, promoting transparency and accountability. By establishing a robust and reliable system for cannabinoid analysis, the industry can build consumer trust, drive market growth, and solidify the European hemp industry’s position in the ever-expanding global market. As the regulatory landscape evolves, efforts to standardize testing and enhance transparency will play a pivotal role in addressing the reported versus measured concentrations of cannabinoids in European hemp products.

## 5. Concluding Remarks and Future Perspectives

The resurgence of hemp cultivation in Europe has been catalyzed by the increased demand in hemp-derived products as well as the less restrictive legislative landscape (wherein changes in laws were driven by public pressure), leading to a flourishing industry with diverse applications. The historical significance of hemp as a valuable resource for textiles, construction, food, and medicine has been revived, showcasing its potential to revolutionize multiple industries in a sustainable and eco-friendly manner. The increasing interest in hemp-derived compounds, particularly CBD, has allowed for new opportunities in the wellness and pharmaceutical sectors. Moreover, research and innovation in hemp science have advanced significantly, providing insights into cannabinoids, terpenes, and their potential therapeutic applications. Navigating the complexities of *Cannabis sativa* L. research presents a host of intriguing challenges, each offering a pathway to deeper insights and progress. For instance, understanding the role of terpenes in shaping the unique traits of different cannabis strains raises questions about their potential interaction with cannabinoid receptors. Exploring the precise functions and interactions of other aromatic compounds, including aldehydes, ketones, alcohols, esters, nitrogen-containing compounds, and phenols, remains an intriguing yet elusive endeavor, ripe for further exploration. The interplay of genetics and environmental factors in influencing cannabinoid and terpene profiles highlights the importance of optimizing cultivation practices to achieve desired outcomes. The hemp industry in Europe faces both challenges and opportunities, with diverse regulations and market acceptance being key factors to navigate.

The future of hemp cultivation and scientific progress in Europe hold immense promise. As research in hemp science continues to advance, deeper insights into minor cannabinoids and their potential therapeutic benefits are anticipated. The use of classical plant breeding techniques as well as genetic engineering (including CRISPR technology) offer exciting possibilities to create novel, innovative hemp varieties with elevated and/or unique cannabinoid and terpene profiles, further unlocking the plant’s potential for diverse industrial and medical applications. However, alongside scientific exploration, it is crucial to navigate ethical considerations, regulatory compliance, and responsible research practices, particularly in breeding and genetic engineering. Collaboration among researchers, cultivators, and policymakers will play a pivotal role in establishing standardized testing protocols and regulations to address the reported versus measured concentrations of cannabinoids in hemp products. The sustainability and eco-friendliness of hemp field cultivation make it a compelling option for industries seeking environmentally conscious solutions. Balancing innovation with sustainability is paramount, calling for the incorporation of practices like water-efficient irrigation, organic farming, and responsible land management to mitigate environmental risks associated with hemp cultivation. As the hemp industry in Europe continues to grow and evolve, responsible practices and regulatory frameworks will be essential to maximize the benefits of this versatile and valuable crop for society and the environment. The importance of clear trade rules and the elimination of cross-border restrictions to facilitate hemp production cannot be overstated. Clear trade regulations ensure smooth operations and promote fair competition within the industry. By removing cross-border restrictions, hemp producers gain access to larger markets, fostering growth and innovation in the sector. By embracing innovative research, sustainable practices, and sound policies, Europe’s hemp industry is poised to help shape a more sustainable future.

## Data Availability

Data used to generate figures in this review were obtained from multiple publicly available sources, namely, USA data from commercially available varieties—Smith, C.J.; Vergara, D.; Keegan, B.; Jikomes, N. The phytochemical diversity of commercial cannabis in the United States. *PLoS ONE* **2022**, *17*, e0267498. PMID: 35588111; Swiss data were obtained from labels of the products and certificates of analysis; European data were obtained from Schafroth, M.A.; Mazzoccanti, G.; Reynoso-Moreno, I.; Erni, R.; Pollastro, F.; Caprioglio, D.; Botta, B.; Allegrone, G.; Grassi, G.; Chicca, A.; et al. Δ9-cis-Tetrahydrocannabinol: Natural occurrence, chirality, and pharmacology. *J. Nat. Prod.* **2021**, *84*, 2502–2510. https://doi.org/10.1021/acs.jnatprod.1c00513. PMID: 34304557.

## Supplementary Materials

The following supporting information can be downloaded at https://www.mdpi.com/article/10.3390/molecules29102397/s1, Table S1: Cannabinoid data for varieties.
